# Acute intercostal pulmonary hernia on postoperative day 5 following uniportal video-assisted thoracoscopic surgery: Successful emergency manual reinsertion case report and literature review

**DOI:** 10.1097/MD.0000000000045420

**Published:** 2025-10-17

**Authors:** Shaoqing Huang, Xu Song, Qiang Shi, Jie Li

**Affiliations:** a Department of Thoracic Surgery, Ningbo No. 2 Hospital, Ningbo, China.

**Keywords:** manual reinsertion, pulmonary hernia, uniportal video-assisted thoracoscopic surgery

## Abstract

**Rationale:**

An intercostal pulmonary hernia is an uncommon condition characterized by protrusion of the pulmonary parenchyma into the thoracic cavity through a compromised or defective chest wall. However, pulmonary hernia management remains debated.

**Patients concerns:**

In this case report, we present a patient who developed an acute pulmonary hernia on the 5th day after uniportal video-assisted thoracoscopic pulmonary surgery. A 57-year-old male patient developed an acute cough accompanied by high fever and reported pain at the left incision site.

**Diagnoses:**

A chest computed tomography scan revealed that the diameter of the hernia sac neck was approximately 4.88 cm, while the maximum diameter of the pulmonary hernia was approximately 6.59 cm.

**Interventions:**

The pulmonary hernia was successfully treated by manual reinsertion.

**Outcomes:**

Follow-up examinations with chest computed tomography and radiography were conducted on the day of manual reinsertion, 3 days later, and 2 weeks after discharge; these assessments indicated that there was no recurrence of pulmonary hernia.

**Lessons:**

This report represents the 1st documented instance of manual reinsertion for acute pulmonary hernia and demonstrates that this therapeutic approach is both safe and feasible. However, a larger sample size and a longer follow-up period are required.

## 1. Introduction

Pulmonary hernia is defined as the protrusion of the lung parenchyma beyond the thoracic cavity resulting from a weakness or defect in the chest wall. Roland reported the earliest documented case of pulmonary hernia in 1499.^[1]^ Subsequently, in 1845, Morel-Lavallee established a classification system based on an analysis of 32 patients admitted for pulmonary hernias, categorizing them into supraclavicular, thoracic, diaphragmatic, and congenital versus acquired hernias according to their anatomical location and underlying etiology.^[2]^ The management of pulmonary hernia remains a subject of debate; while most researchers advocate for conservative treatment only, some authors contend that surgical intervention may be necessary for pulmonary hernia.^[3]^ In this report, we present the 1st documented case of an acute pulmonary hernia following pulmonary surgery that was effectively managed through manual reinsertion.

## 2. Case presentation

**Figure 1.**
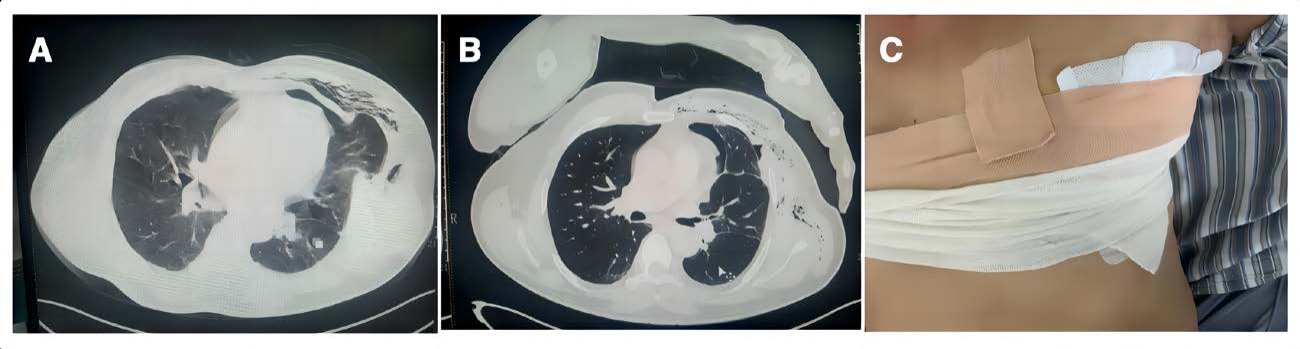
(A) Pulmonary hernia at the 4th intercostal space on the axillary front; (B) pulmonary hernia returning to the thoracic cavity after manual reinsertion; (C) compression bandaging of the chest wall after manual reinsertion.

A 57-year-old male patient with a history of smoking was admitted to the hospital following the discovery of a pulmonary nodule 1 week prior. His lung function was normal and he had no significant medical history. The patient underwent uniportal video-assisted thoracoscopic surgery, during which wedge resection of the left upper lobe and lymph node dissection were performed through the 4th intercostal space along the axillary frontline. Pathological examination revealed adenocarcinoma in situ in the lung. Postoperatively, a 26 French thoracic tube was placed for drainage of pleural effusion and air. A standard perioperative management plan for thoracic surgery was implemented, leading to the removal of the thoracic tube on postoperative day 4. A chest radiograph conducted on the morning of day 5 showed satisfactory results; however, in the afternoon, the patient developed an acute cough accompanied by high fever and reported pain at the left incision site. Physical examination indicated a rash and shock-like sensations upon coughing at this site. Immediate assessments included pathogen detection, inflammatory marker evaluation, and chest computed tomography (CT). Chest CT revealed a pulmonary hernia in the 4th intercostal space along the axillary front line. Blood tests revealed normal white blood cell counts and elevated C-reactive protein levels elevated to 36.56 mg/L, while procalcitonin remained within normal limits. The patient received antipyretic treatment alongside penicillin antibiotic therapy to manage the infection, and manual reinsertion of the pulmonary hernia was performed as follows: upon coughing, we palpated around the location of herniation while instructing him to take deep breaths; simultaneously applying pressure from peripheral areas towards the center around the incision until the sensation subsided; thereafter, compression with gauze and elastic bandage ensuring tightness sufficient for one finger insertion. At this stage, the patient reported a significant reduction in the pain in the left chest. Subsequently, chest CT was performed to confirm that the pulmonary hernia had returned to its anatomical position within the thoracic cavity (Fig. 1). On the 6th postoperative day, we performed tests for COVID-19 and influenza. The results indicated a positive nucleic acid test for the type A influenza virus. The patient received oral antiviral treatment with marvaroxavir and exhibited a satisfactory recovery. On the 8th postoperative day, another chest CT scan was performed, which revealed that the incision defect on the chest wall had been successfully repaired. The patient was discharged from the hospital on the 9th postoperative day. Two weeks post-discharge, the patient returned for a follow-up chest radiograph, which confirmed that there had been no recurrence of the pulmonary hernia (Table 1).

**Table 1:** Timeline of important events.

Time line	Event
Operation day	–
Four days after the operation	Remove the thoracic tube
Five days morning after the operation	Pulmonary hernia (−)
Severe cough (afternoon)	Pulmonary hernia (+)
Manual reinsertion (afternoon)	Pulmonary hernia (−)
Eight days after the operation	Pulmonary hernia (−)
Two weeks after discharge	Pulmonary hernia (−)

(+) = it indicates a pulmonary hernia; (−) = it indicates there is no pulmonary hernia.

## 3. Discussion and conclusion

Pulmonary hernia is an exceedingly rare condition that is often asymptomatic or is associated with chest pain. Congenital and acquired pulmonary hernias account for approximately 20% and 80% of the cases, respectively.^[4]^ Acquired pulmonary hernias can be classified into traumatic, spontaneous, and pathological types.^[3]^ Factors such as smoking and obesity are known to induce spontaneous pulmonary hernias.^[5]^ Additionally, acquired pulmonary hernias may develop due to increases in intrathoracic pressure resulting from activities such as coughing, sneezing, heavy lifting, or abnormal movements, alongside rib fractures.^[1,6,7]^ Diagnosis primarily relies on chest CT and chest radiography.^[8]^ Complications associated with untreated pulmonary hernias include pneumonia, pneumonitis, pleural scarring, atelectasis, hemoptysis, strangulation, and impaired pulmonary function.^[9–11]^ Thoracotomy has been recognized as a potential cause of acquired pulmonary hernia, and previous reports indicate that intercostal pulmonary hernias typically manifest between 3 months and 8 years after post-thoracotomy^[12]^; however, the present case developed a pulmonary hernia on the 5th postoperative day, an occurrence that diverges from prior findings. Chest CT remains the gold standard for diagnosing pulmonary hernia, whereas physical examination may reveal crepitations upon palpation.^[13]^ Early surgical intervention is recommended for anterior chest wall pulmonary hernias; nevertheless, the necessity of surgical repair remains a subject of debate.^[12]^

**Figure 2.**
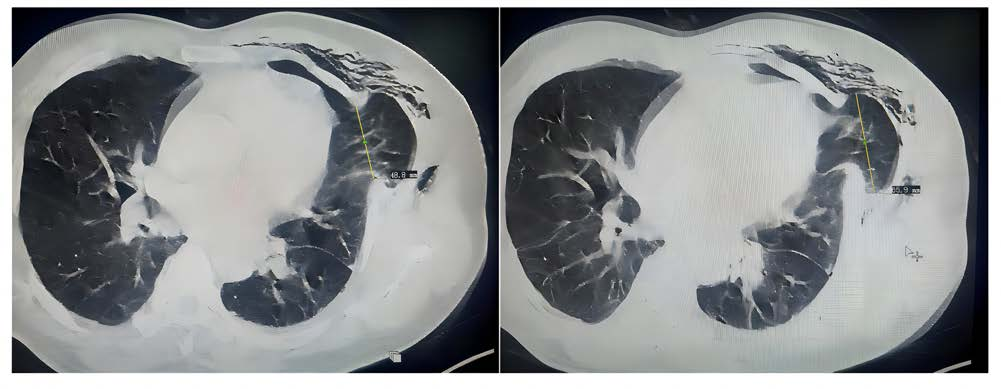
Pulmonary hernia measurements.

In this case, the patient underwent uniportal video-assisted thoracoscopic surgery, which resulted in a muscle defect located in the left 4th intercostal space. Following removal of the patient’s chest tube, the intercostal defect space was enlarged. Severe coughing leads to increased pressure within the pleural cavity, causing muscle tears at the incision site. This ultimately results in the protrusion of residual lung tissue through the 4th intercostal space and subsequent shifting, culminating in a pulmonary hernia. A chest CT scan revealed that the diameter of the hernia sac neck was approximately 4.88 cm, while the maximum diameter of the pulmonary hernia was approximately 6.59 cm (Fig. 2). The pulmonary hernia was promptly identified; given that no ischemic necrosis was present, it could be repaired via manual reinsertion techniques. Ultimately, we successfully made a clinical decision to manage an acute pulmonary hernia through manipulation methods. We recommend retracting the hernia during deep inhalation by expanding both the thoracic cavity volume and enhancing the negative pressure within it, which facilitates effective retraction of the hernia. Additionally, when patients cough, any associated shock from lung displacement combined with chest pain should prompt reevaluation to ensure complete resolution of pulmonary herniation. Follow-up chest CT was performed to confirm successful reinsertion of the pulmonary hernia.

In light of the ongoing prevalence of COVID-19 in certain areas, which can lead to severe coughing,^[14]^ we conducted tests for both COVID-19 and influenza virus infections. Appropriate antiviral medication was administered based on the results obtained. Following treatment, the patient’s cough significantly improved, contributing to the overall success of our intervention.

However, this study has several limitations. First, the sample size consisted of only 1 case, which limits the statistical power and generalizability of the findings. Second, the follow-up duration was relatively short; therefore, the long-term outcomes, including the potential for recurrence of pulmonary hernia, remain uncertain. These factors should be considered when interpreting the results.

In conclusion, we report a rare perioperative complication following uniportal video-assisted thoracoscopic surgery for pulmonary hernias. For the 1st time, we demonstrated that manual reinsertion of a pulmonary hernia in an emergency setting is both safe and feasible. This article also discusses critical aspects related to the manual reinsertion procedure for pulmonary hernias.

## Author contributions

**Conceptualization:** Shaoqing Huang.

**Funding acquisition:** Shaoqing Huang.

**Methodology:** Xu Song.

**Project administration:** Shaoqing Huang.

**Writing – original draft:** Shaoqing Huang.

**Writing – review & editing:** Shaoqing Huang, Qiang Shi, Jie Li.
